# Human Skeletal Muscle Disuse Atrophy: Effects on Muscle Protein Synthesis, Breakdown, and Insulin Resistance—A Qualitative Review

**DOI:** 10.3389/fphys.2016.00361

**Published:** 2016-08-25

**Authors:** Supreeth S. Rudrappa, Daniel J. Wilkinson, Paul L. Greenhaff, Kenneth Smith, Iskandar Idris, Philip J. Atherton

**Affiliations:** Division of Medical Sciences and Graduate Entry Medicine, School of Medicine, MRC-Arthritis Research UK Centre for Musculoskeletal Ageing Research, Royal Derby Hospital, University of NottinghamDerby, UK

**Keywords:** skeletal muscle, disuse, immobilization, protein metabolism, diabetes

## Abstract

The ever increasing burden of an aging population and pandemic of metabolic syndrome worldwide demands further understanding of the modifiable risk factors in reducing disability and morbidity associated with these conditions. Disuse skeletal muscle atrophy (sometimes referred to as “simple” atrophy) and insulin resistance are “non-pathological” events resulting from sedentary behavior and periods of enforced immobilization e.g., due to fractures or elective orthopedic surgery. Yet, the processes and drivers regulating disuse atrophy and insulin resistance and the associated molecular events remain unclear—especially in humans. The aim of this review is to present current knowledge of relationships between muscle protein turnover, insulin resistance and muscle atrophy during disuse, principally in humans. Immobilization lowers fasted state muscle protein synthesis (MPS) and induces fed-state “anabolic resistance.” While a lack of dynamic measurements of muscle protein breakdown (MPB) precludes defining a definitive role for MPB in disuse atrophy, some proteolytic “marker” studies (e.g., MPB genes) suggest a potential early elevation. Immobilization also induces muscle insulin resistance (IR). Moreover, the trajectory of muscle atrophy appears to be accelerated in persistent IR states (e.g., Type II diabetes), suggesting IR may contribute to muscle disuse atrophy under these conditions. Nonetheless, the role of differences in insulin sensitivity across distinct muscle groups and its effects on rates of atrophy remains unclear. Multifaceted time-course studies into the collective role of insulin resistance and muscle protein turnover in the setting of disuse muscle atrophy, in humans, are needed to facilitate the development of appropriate countermeasures and efficacious rehabilitation protocols.

## Introduction

Skeletal muscle tissue represents the largest protein/amino acid (AA) reservoir in the human body (Bonaldo and Sandri, 2013). Skeletal muscles are not only crucial for locomotion but also represent the body's largest metabolically active tissue, glucose disposal site, and fuel reservoir for other organs in fasting and pathological conditions (i.e., hepatic supply of amino acids for gluconeogenesis). Loss of muscle mass occurs with many common illnesses (Evans, 2010) including cancers (Stephens et al., 2010), renal/heart failure (Gordon et al., 2013), sepsis (Gordon et al., 2013), muscle genetic diseases (Sandri, 2010), and neurodegenerative disorders (Verdijk et al., 2012). Muscle atrophy also occurs in otherwise healthy individuals under situations of reduced neural input—such as during immobilization, e.g., due to leg casting for fractures (Phillips et al., 2009), bed-rest, spinal cord injury (Castro et al., 1999) and during “aging” (i.e., sarcopenia; Evans and Lexell, 1995). The main environmental determinants of muscle mass in adulthood are exogenous essential amino acids (AA) (needing to be acquired through dietary protein intake), Newton's gravity and locomotion (DeFronzo and Tripathy, 2009). Indicative of this, lack of energy intake during starvation (Rennie et al., 2010), inactivity (Wall et al., 2013), spaceflight (Vandenburgh et al., 1999), or limb immobilization (Phillips et al., 2009) all lead to a reduction in muscle cross sectional area (CSA), an associated loss of function, and muscle insulin resistance. Crucially, loss of muscle mass is associated with greater morbidity and mortality (Sasaki et al., 2007), reduced independence, especially in older populations (Leenders et al., 2013) and this is accelerated in type 2 diabetes (Leenders et al., 2013).

### Overview of disuse atrophy, countermeasures, and muscle metabolism

Disuse atrophy is often referred to as “simple atrophy” in that atrophy is intrinsic to the muscle(s) specifically exposed to disuse; that is, disuse atrophy is not a systemic condition. Countermeasures for disuse atrophy are limited but include forms of mechanical loading (Wilkinson et al., 2008) such as exercise/electrical stimulation (Wall et al., 2012), passive physiotherapy (Fowles et al., 2000) and harnessing the adjunct anabolic effects of protein nutrition (Churchward-Venne et al., 2012b). Ascertaining an understanding of the mechanisms of disuse atrophy—particularly in relation to the regulation of muscle protein synthesis (MPS) and breakdown (MPB)—is important for designing countermeasures or rehabilitation protocols (Reggiani, 2015). Furthermore, despite accumulating evidence that physical inactivity plays a causative role in development of non-communicable diseases such as obesity, insulin resistance, type 2 diabetes and dyslipidemia (Atherton et al., 2016), the mechanistic role of muscle insulin resistance (IR) in driving muscle atrophy in the context of “simple disuse” remains unclear (Atherton et al., 2016). This review will focus on identifying different models of human disuse atrophy, the degree of muscle and strength loss and the regulation of muscle protein turnover and muscle IR. Future translational studies to mitigate disuse atrophy will rely upon robust evidence being present in humans of active mechanisms (often of putative mechanisms that have been pre-identified in animals). As such, this review will focus mainly on current evidence from clinical studies.

### The impacts of experimental disuse on muscle mass and strength loss in humans

A plethora of clinical studies have investigated the degree of muscle loss in humans exposed to disuse. The most frequent employed models to study disuse atrophy in humans are unilateral limb suspension (ULLS) using a knee brace or cast, and bed rest; other scenarios include spinal cord injury and spaceflight. In terms of muscle mass, the observed rate of decline in muscle size (CSA) for each day of ULLS in knee extensors was ~0.40% and ~0.36% for plantar flexors following 42 days of unloading (Hackney and Ploutz-Snyder, 2012). Other studies have demonstrated losses of muscle strength and mass early on in disuse, i.e., 5 days of cast immobilization lead to ~3.5% reductions in quadriceps CSA and ~9% in strength (Dirks et al., 2014). This had progressed to ~8% reductions in CSA and ~23% reductions in strength by 14 days (Wall et al., 2013). Additionally, Suetta et al. reported ~10% reductions in myofibre area and ~13% decreases in strength after just 4 days progressing to ~20% reductions in myofibre area and strength after 14 days of ULLS (Suetta et al., 2012, 2013). A further study reported decreases in mid-thigh CSA of 11% following 28 days of bed rest (Brooks et al., 2008). Lastly, a study by Castro et al. showed muscle CSA to be ~45% less compared to able-bodied controls 6-weeks after complete spinal cord injury (Castro et al., 1999). Adding to the above constellation, Gibson et al. studied men who were immobilized following tibial fracture (thus having 6-weeks of casting) and reported reductions in quadriceps CSA of ~17%. Furthermore, Alkner et al. reported that 90 days bed rest led to ~10 and ~16% reductions in quadriceps and triceps surae mass after 29 days, with rates of weekly loss slowing during the last 2 months to roughly half that observed during the first month (Alkner and Tesch, 2004). Finally, muscle CSA decreased by ~5% (de Boer et al., 2007; Glover et al., 2010) at 14 days and 10%, at 23 days, i.e., 0.5% day following ULLS (de Boer et al., 2007). Collectively, these studies indicate a varying degree of rates of disuse muscle atrophy, depending on the duration and nature of immobilization but also measurement techniques, i.e., MRI/DXA/ultrasound/ myofibre CSA; however, it appears atrophy occurs more rapidly in first 3–14 days of unloading and eventually reaching a nadir where further loss of muscle occurs at a slower rate despite continued unloading of muscle (Bodine, 2013).

Differences in the rate of muscle atrophy have also been observed according to different muscle and fiber types as well as the mode of immobilization. For example, after prolonged disuse (~180 days of space flight), loss of fiber size and force was reported in the soleus and gastrocnemius muscles with the order of atrophy (greatest-least) being: soleus type I > soleus type II > gastrocnemius type I > gastrocnemius type II (Fitts et al., 2010). Similar effects of disuse on fiber type following 35 days of bed rest was reported in the vastus lateralis (VL) muscle, i.e., the loss of fiber CSA was greater in type 1 than type II fibers (Brocca et al., 2012). Conversely, muscle fiber type specificity has not been observed in other studies (Bamman et al., 1998; Trappe et al., 2008; Hvid et al., 2010) where duration of immobilization was shorter (<14 days). It is notable that, these studies have mainly focused on a single muscle with muscle biopsy taken from a single site in the periphery of the muscle. This is relevant because muscles do not atrophy uniformly across the entire length of a single muscle (Miokovic et al., 2012), with differential atrophy across different muscles being observed following 27–60 days head-down-tilt bed rest. The investigators also reported that the posterior calf muscles atrophied faster than the knee extensor muscles (Vastus Lateralis) and ankle flexors (Tibialis anterior). In another study where multiple muscles were examined using MRI over 43 days disuse in the form of ankle immobilization, the greatest rate of muscle loss was observed in soleus and medial gastrocnemius muscle followed by lateral gastrocnemius and tibialis anterior (Psatha et al., 2012). The aetiology driving distinct fiber type, and individual muscle atrophy susceptibility is poorly defined.

### Regulation of skeletal muscle mass in ambulated and unloaded human muscle

#### Ambulated regulation of MPS and MPB

Skeletal muscle mass is regulated by the balance between MPS and MPB. Nutrients (i.e., AA) and nutrient derived hormones (i.e., insulin) play a crucial role in regulating the balance between MPS and MPB. In ambulated humans, intake of dietary protein stimulates MPS due to the essential amino acids (EAA) components of proteins (Atherton and Smith, 2012). These anabolic responses are dose-dependent and saturable; at a maximal stimulus, rates of MPS increase ~200–300% for a period of ~2 h following ~20 g protein (Cuthbertson et al., 2005; Atherton and Smith, 2012). In contrast, insulin released following intake of dietary protein and/or CHO, is neither necessary nor sufficient to stimulate MPS (Greenhaff et al., 2008). Reflecting this, the anti-catabolic effects of insulin upon MPB was not recapitulated via AA infusions when insulin concentrations were clamped at 5 μU.ml^−1^ (post absorptive; Greenhaff et al., 2008). Instead, insulin concentrations of just 15 IU/ml (3 × post absorptive) are sufficient to maximally suppress MPB (Wilkes et al., 2009). This anti-catabolic effect of insulin acting on MPB was confirmed in a recent systematic review and meta-analysis of 44 human studies, which concluded insulin did not significantly affect MPS but has a crucial role in reducing MPB (Abdulla et al., 2016). Therefore, while EAA's stimulate MPS, insulin suppresses MPB (and stimulates muscle glucose uptake). On the basis, EAA and insulin are so vital in maintaining muscle metabolic homeostasis, failure of these mechanisms inevitably leads to skeletal muscle atrophy and IR.

#### Impact of disuse on MPS and anabolic pathways

Disuse of human skeletal muscle alters muscle metabolism dynamics (Mallinson and Murton, 2013). For instance, early work by Gibson et al. showed that young men exposed to ULLS exhibited ~30% slower rates of fasted state MPS compared to the contralateral non-immobilized limb (Gibson et al., 1987). Subsequent studies confirmed reductions in MPS; for instance, ~50% reductions in MPS following 2-weeks of bed rest (Ferrando et al., 1996; Paddon-Jones et al., 2004) and ULLS with braces/casting (Glover et al., 2008). In further agreement with this, Kortebein et al. reported ~30% reductions in post-absorptive MPS during a 24 h period in older adults after 10 days of bed rest (Kortebein et al., 2007). Crucially, blunting of MPS in response to muscle unloading is not restricted to fasted periods. Glover et al. reported that ULLS led to ~27% reductions in postprandial rates of MPS at both low and high doses of AA infusions (Glover et al., 2008). Similarly, Drummond et al. reported that 7 days of bed rest blunted fed state MPS following EAA ingestion (Drummond et al., 2012). Similarly, 14 days of ULLS led to a ~30% reduction in MPS after ingestion of 20 g dietary protein (Wall et al., 2013). On this basis, available evidence strongly supports the notion that skeletal muscle atrophy in humans during a period of disuse is driven by blunting of both postabsorptive and postprandial MPS (Rennie et al., 2010; Wall et al., 2013).

The mTOR (mechanistic target of rapamycin) pathway is the major signal transduction network “hub” involved in the regulation of mRNA translation. Cell and rodent based research suggests this system senses important stimuli responsible for the regulation of MPS, i.e., (1) insulin and insulin-like growth factor-1 (IGF-1) through IRS (insulin receptor substrate) and PI3K (Phosphatidylinositol-3) pathways (Wackerhage and Ratkevicius, 2008); (2) AA through leucyl-tRNA/Rag-mTORc1 pathways (Wackerhage and Ratkevicius, 2008), (3) Energy stress through AMPK-eukaryotic elongation factor (AMP activated protein kinase/ eEF2) pathway (Wackerhage and Ratkevicius, 2008), and (4) mechanical stress, e.g., through mechano-sensory pathways (Wackerhage and Ratkevicius, 2008). However, the impact of these factors in the regulation of MPS in human remains unclear. For example, under ambulated conditions, one of the mTOR upstream effector, the class III PI3K hVps34 (human vacuolar protein sorting-34) was shown to be inhibited in amino acid starved state (basal condition) and increased activity of this protein appears to be concomitant with increased S6K1, suggesting it to be key player in mTOR signaling pathway (Dickinson and Rasmussen, 2011). Furthermore, intake of dietary protein typically leads to increase in phosphorylation status of class III PI3K hVps34 (Dickinson and Rasmussen, 2011), mTOR and its downstream substrates regulating mRNA translation, such as p70^S6K^ (Drummond et al., 2012). However, in response to disuse, blunted phosphorylation of mTOR and p70^S6K^ was shown after 2-weeks of immobilization while, no significant increases in phosphorylation of mTOR and p70^S6K^ were noted after immobilization (Wall et al., 2013). In contrast, others have reported no decreases in Akt/mTOR signaling despite reductions in MPS (de Boer et al., 2007; Wackerhage and Ratkevicius, 2008). It is worthwhile noting that peak stimulation of signaling proteins (mTOR and p70^S6K^) occurs 1–2 h after protein intake while many studies have muscle biopsies taken 3–6 h after protein intake when the response would be attenuated or perhaps absent (Glover et al., 2010; West et al., 2011; Churchward-Venne et al., 2012a; Drummond et al., 2012). Further studies are needed with frequent biopsy sampling to fully determine a role for deactivation in mTORC1-related signaling networks (or indeed other putative mechanisms) regulating depressions in post-absorptive and post-prandial MPS.

#### Impact of disuse on MPB and catabolic pathways

**Figure 1:**
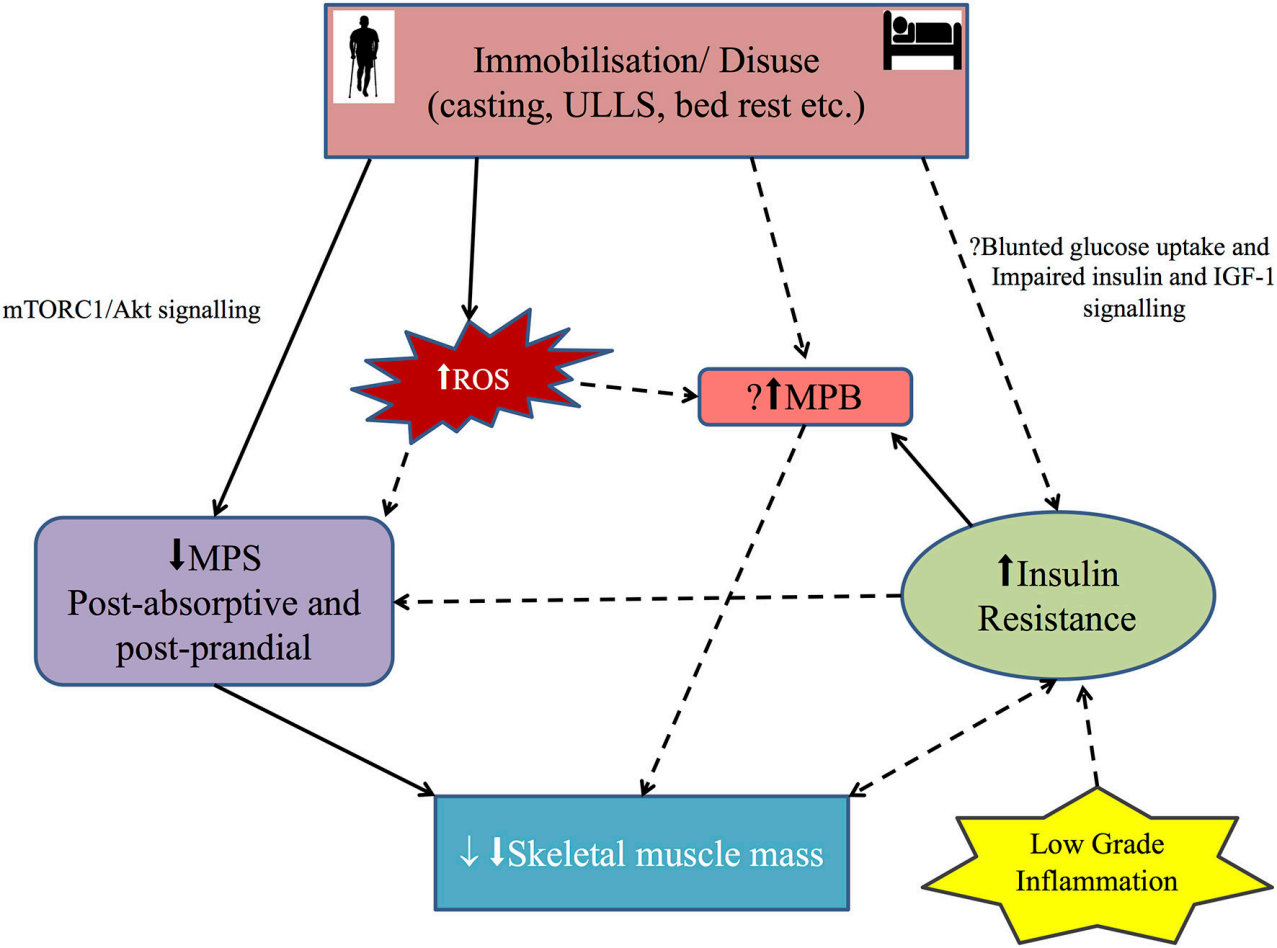
**Diagrammatic representation of the main mechanisms involved in disuse skeletal muscle atrophy in humans:** Immobilization/disuse reduces both postabsorptive and post prandial muscle protein synthesis (MPS) via the mammalian target of rapamycin (mTORC1) and Akt signaling. The role of MPS, muscle protein breakdown (MPB) and insulin resistance (IR) in simple disuse atrophy remain poorly defined in humans. So the role of insulin resistance and MPB in the setting of disuse atrophy needs further evaluation. Inflammation probably leads to IR. Recently, reactive oxygen species (ROS) has been implicated in development of muscle atrophy in disuse setting, but the mechanism in human remains putative. Solid arrow shows positive association and broken arrow shows putative association. See text for more details.

In contrast to the recognized deficits in MPS during immobilization, the role of MPB in disuse atrophy is less clear. This is partly confounded by the technical and clinical challenges in measuring *in vivo* rates of MPB (e.g., arterio-venous balance methods to measure rate of appearance i.e., breakdown and pulse chase stable isotope approaches to measure fractional breakdown rates; Kumar et al., 2009; Atherton and Smith, 2012; Atherton et al., 2016). In the only study to our knowledge to measure this, Symons et al. demonstrated no increase in MPB in his study of healthy young volunteers exposed to 21 days of microgravity setting using a bed rest model (Symons et al., 2009). Although not directly quantifying MPB, Wall et al. reported that muscle free tracer enrichment over 4-h post prandial period was >3 fold higher after immobilization (Wall et al., 2013). Since MPS has consistently been shown to be reduced with immobilization, a likely explanation is that MPB is actually reduced (rather than increased) and hence the less unlabeled phenylalanine efflux was diluting the muscle free labeled tracer (L-(ring-^2^H_5_) phenylalanine pool (Wall et al., 2013). Alternatively, an accumulation of free tracer could simply be explained by established “anabolic resistance,” i.e., where a failure of AA incorporation into the muscle lead to its accumulation (Glover et al., 2008; Phillips et al., 2009; Rennie, 2009; Wall et al., 2013). Nevertheless, regardless of the driving force behind muscle atrophy (i.e., disuse, aging, cancer, organ failure), blunted postabsorptive and postprandial MPS (Figure 1; anabolic resistance) seem to be the major drivers of disuse atrophy—rather than increases in MPB. Nonetheless, more work is needed across the time-course of unloading to verify this.

**Figure 2:**
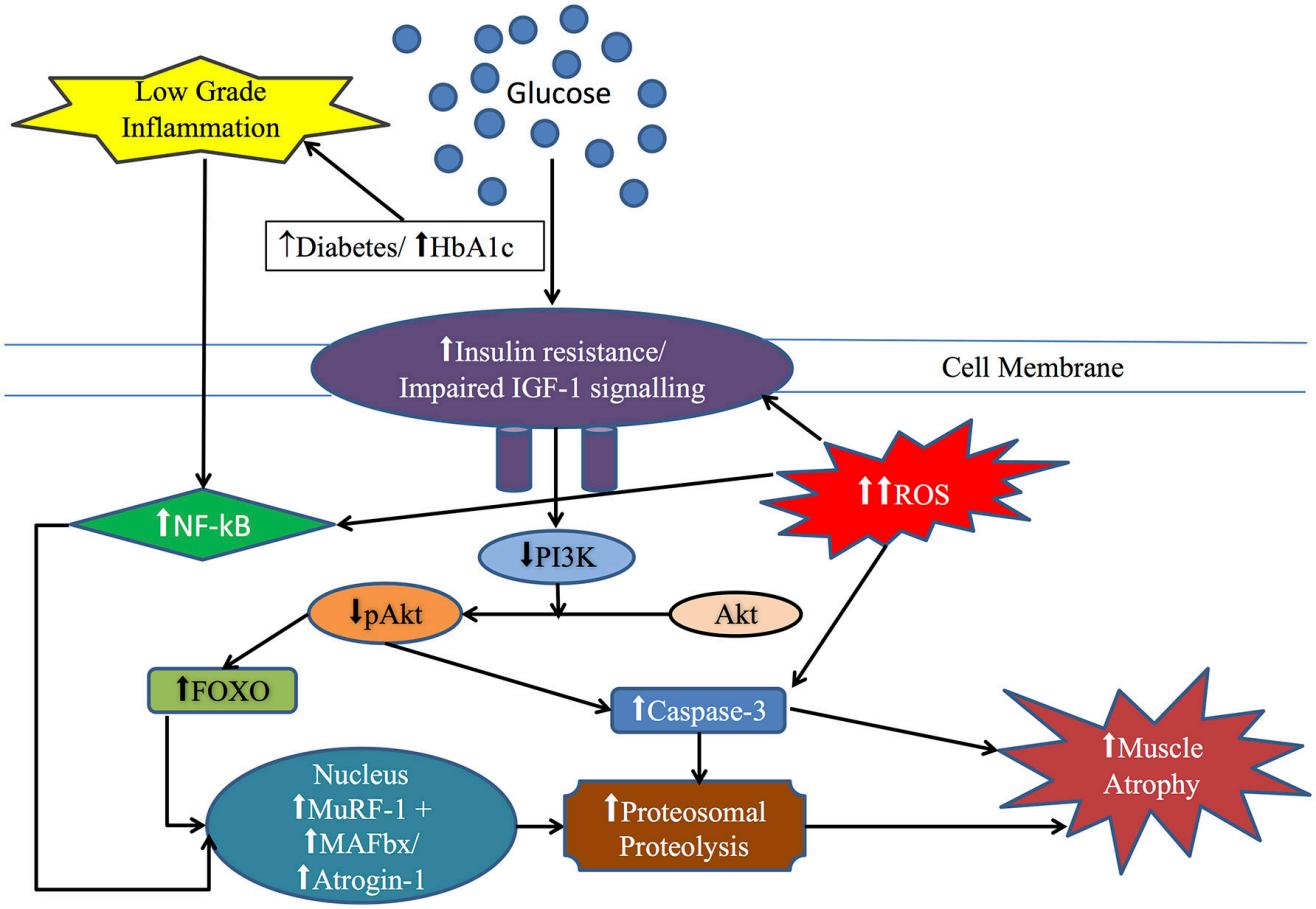
**Diagrammatic representation of the overlap between insulin signaling pathway, reactive oxygen species (ROS), inflammatory cytokine such as NF-κB and ubiquitin-proteasome system in insulin resistant (IR) states particularly diabetes:** In IR state, PI3K activity is decreased, leading to decreased activity of Akt, which in turn release the inhibition of FOXO and caspase-3 resulting in elevation of muscle ring finger-1 (MuRF-1) and muscle atrophy F-box (MAFbx/atrogin-1) finally leading to increased proteolytic activity. Also, ROS and low grade inflammation via NF-κB pathway lead to muscle atrophy. See text for more details.

In terms of the molecular regulation of MPB, the ubiquitin proteasome system (UPS; Lecker et al., 2006) supplemented by lysosomal and calcium activated calpain (ATP–independent) and caspase dependent cleavage of actinomyosin complexes (Glover et al., 2010) are the major catabolic pathways in muscle. The identification of the “atrogenes” as genes that are uniformly upregulated irrespective of the atrophy stimulus (e.g., denervation, disuse, thermal injury) has received much attention as key drivers of atrophy programming (Bodine et al., 2001a; Jones et al., 2004; Milan et al., 2015). This led to members of the Forkhead Box (Fox) O family (Fox1, 3, and 4) being identified as downstream targets of Akt pathway (Figure 2) and as the main transcription factors regulating MAFbx/atrogin-1expression (Sandri et al., 2004). In terms of disuse atrophy, mRNA expression of two E3 ubiquitin ligases was initially found to be crucial in immobilization, unloading and denervation induced muscle atrophy (Bodine et al., 2001b). These genes, MuRF-1 (Muscle Ring Finger-1), and MAFbx/atrogin-1 (Muscle Atrophy F-box), are expressed in skeletal muscle at low levels but rapidly induced in response to unloading (Bodine et al., 2001b). In humans, after 5 days (Dirks et al., 2014) and 2 weeks (Jones et al., 2004) of immobilization, MAFbx and MuRF1 mRNA were reported to be elevated. Nonetheless, while their expression is thought to be regulated by transcription factors such as FOXO1, FOXO3a, (Sandri et al., 2004) and NFκβ (p50 and Bcl-3)(Wu et al., 2011), no increase in mRNA expression in FOXO's were noted after 4 or 14 days of immobilization (Suetta et al., 2012). De Boer et al reported the expression of MuRF-1 but not MAFbx mRNA was increased after 10 days of ULLS (Jones et al., 2004; de Boer et al., 2007), while both had decreased by 21 days (de Boer et al., 2007). Furthermore, increases in UPP components (particularly UBE2E) were up-regulated 48 h following instigation of ULLS (Urso et al., 2006). In contrast, a recent study by Brocca et al. found that muscle atrophy following ULLS found no change in mRNA expression of ubiquitin-proteasome and autophagy systems (Brocca et al., 2015). Some work has been done in relation to autophagy (and calpain-signaling) in relation to human disuse (Jones et al., 2004). Autophagy is responsible for removing unfolded, damaged and dysfunctional proteins and organelles via the formation of autophagosomes for degradation by lysosomes (Sandri, 2010). Interestingly, up-regulation of autophagy markers such as Beclin-1 suggested increased autophagosome formation and hence a higher activity of the macro-autophagy by 24 days of bed rest; nonetheless, other autophagy markers such as P62, LC3II/I ratio, and cathepsin-L were not up-regulated (Brocca et al., 2012). While details of the pathways discussed above are outside the scope of this review, the readers are referred to reviews by Bonaldo and Sandri (2013) and Sandri (2010). What is clear however is that without more clinical studies with time-course acquisition, including dynamic measures of MPB in tandem with molecular markers spanning different proteolytic systems, no firm conclusions can be made surrounding the mixed results regarding whether existing molecular data suggest MPB is increased, decreased or unchanged in response to disuse in humans.

In addition to the anabolic and catabolic pathways described above, emerging evidence indicates that disturbed redox signaling may also be an important regulator of MPS and MPB in muscle disuse atrophy (Powers et al., 2012; Zuo and Pannell, 2015). Oxidative injury has been shown to occur in muscle fibers during periods of disuse in locomotor skeletal muscles (Min et al., 2011) and in non-load bearing muscle such as the diaphragm during prolonged mechanical ventilation (Kavazis et al., 2009). After 35 days of bed rest, vastus lateralis muscle showed ~18% muscle fiber atrophy and increased protein carbonylation (Dalla Libera et al., 2009). Furthermore, an inverse linear relationship was observed between normalized levels of protein oxidation and muscle fiber CSA (Dalla Libera et al., 2009). An analysis of gene expression showed up-regulation of pathways involved in the oxidative stress response (increase in mRNA for stress response gene heme oxygenase-1) following 48 h of unilateral lower leg suspension (ULLS; Reich et al., 2010). Conversely, in a limb immobilization human model with ~5.7% muscle and 11.8% muscle fiber loss after 14 days of immobilization, no increase in lipid peroxidation and protein oxidation in vastus lateralis was observed (Glover et al., 2010). Although information on oxidative stress and potential mechanisms explaining proteolysis in disuse human muscle is still sparse (Pellegrino et al., 2011), these findings support the extensive evidence available from animal studies that oxidative stress inhibits MPS (Powers et al., 2011a) and increases muscle MPB (via increased gene expression of key proteins involved in the proteolytic pathways such as autophagy, calpains and proteosomes; activation of both calpain and caspase-3 and possibly by modification of myofibrillar proteins which enhances their susceptibility to proteolytic processing; Powers et al., 2011a). Interaction between ROS and insulin signaling pathway has also been described, i.e., ROS may regulate Insulin growth factor-1 (IGF-1) signaling either positively or negatively depending on the amount of ROS produced (Papaconstantinou, 2010). Low levels of endogenous ROS due to their reversible oxidative inhibition of protein tyrosine phosphatases induces phosphorylation of tyrosine residue on the insulin receptor and its substrates triggering IGF-1 signaling (Bashan et al., 2009). In contrast, the IGF-1 signaling pathway is inhibited by higher levels of ROS and recent evidence suggests ROS down regulates the IGF-1 cascade and induces insulin resistance (Bashan et al., 2009; Figure 2). For detailed discussion of the signaling pathways linking ROS and muscle atrophy, the interested reader is referred to recent reviews on oxidative stress and disuse muscle atrophy (Pellegrino et al., 2011; Powers et al., 2011b, 2014; Zuo and Pannell, 2015).

#### Impact of disuse on muscle IR and links to muscle mass in persistent IR states

Insulin-mediated glucose uptake is also blunted with muscle disuse (Mikines et al., 1991; Biensø et al., 2012); that is, unloaded muscle becomes IR. This IR can be observed at a whole-body level following bed-rest, but is most apparent at the muscle level across the physiological range of insulin concentrations under clamp conditions (Mikines et al., 1991). Recently, a 1 week bed-rest study in young males by Dirks et al. revealed reduced muscle mass (~1.4 kg lean tissue and ~3% quadriceps CSA) and whole-body insulin sensitivity (~29%)(Dirks et al., 2016). Thus, disuse lowers MPS, induces anabolic resistance to nutrients and impairs insulin-mediated muscle glucose uptake—even in healthy adults (Fink et al., 1983).

The role of IR in driving muscle atrophy however is poorly defined. Evidence from large cross-sectional and longitudinal studies reports accelerated loss of muscle mass in individuals with persistent IR (i.e., people with Type 2 Diabetes), perhaps pointing to mechanistic links. For instance, declines in muscle mass were inversely related to duration of diabetes or HbA1c (Park et al., 2007, 2009; Kalyani et al., 2013) and attenuated with insulin sensitizers (Kuo et al., 2009). Human muscle tissue accounts for 80% of glucose uptake after food ingestion and insulin resistance (HOMA-IR) is associated with reduced quadriceps muscle strength (Kalyani et al., 2013; Leenders et al., 2013), power (Kalyani et al., 2013) and muscle mass (Leenders et al., 2013) in humans. Approximately a 50% more rapid decline in knee extensor strength has been observed in older patients with type 2 diabetes compared with patients without diabetes over a 3 year period, suggesting that decreased muscle strength may be accelerated in type 2 diabetes (Park et al., 2007). In a further study, Volpato et al. reported differences in walking speed, muscle strength, power and muscle quality between individuals with and without diabetes were independent of co-existing peripheral motor neuropathy or peripheral vascular disease, suggesting a direct effect of diabetes *per se* on muscle performance (Volpato et al., 2012). These findings are important because in catabolic conditions such as diabetes, atrophy in combination with reduced activity decrease quality of life and increase mortality (Zinna and Yarasheski, 2003). Yet despite clear evidence linking accelerated muscle loss in diabetes compared to non-diabetes, studies investigating the direct effect of immobilization on muscle protein turnover in patients with diabetes compared to those without diabetes are scant. Furthermore, clear distinction between Type 1 and Type 2 diabetes needs to be made when investigating patients with diabetes. This is because Type 1 diabetes is a condition with severe depletion of energy stores and reduced mitochondrial function resulting in accelerated muscle protein loss (Hebert and Nair, 2010), which can be reversed by insulin replacement (Workeneh and Bajaj, 2013). In contrast, muscle loss, whilst accelerated in type 2 diabetes, is unaffected by insulin treatment (Workeneh and Bajaj, 2013), possibly due to IR. Hence, skeletal muscle mass loss whilst common, appears to occur less predictably and to varying degree in Type 2 diabetes compared with Type 1 diabetes (Workeneh and Bajaj, 2013). Collectively, these data are consistent with the notion that diabetes causes muscle mass loss possibly due to mechanisms driving muscle IR, however there is lack of data regarding the effects of immobilization or disuse on muscle mass in individuals with diabetes.

The mechanistic regulation of muscle IR in driving muscle atrophy in the setting of “simple disuse” remains vague (Atherton et al., 2016). Early human studies by Shulman et al. showed that, under steady state plasma concentration of both glucose and insulin mimicking postprandial conditions, the mean rate of muscle glycogen synthesis accounted for most of the whole body glucose uptake and virtually all of non-oxidative glucose metabolism in both healthy and diabetic subjects (Shulman, 2000), with defects in muscle glycogen synthesis playing a major role in causing insulin resistance in type 2 diabetes (Shulman, 2000). This may be explained by defects in the insulin receptor substrate (IRS)-1/phosphatidylinositol (PI) 3-kinase pathway, leading to reduced glucose uptake and utilization in insulin target tissues (Draznin, 2006). Free fatty acids induce muscle IR by inhibiting glucose transport/phosphorylation and reductions in both the rate of muscle glycogen synthesis and glucose oxidation (Roden et al., 1996). Additionally, many other mechanisms have been postulated to explain free fatty acid-induced muscle IR, including the Randle cycle, oxidative stress, inflammation and mitochondrial dysfunction (Martins et al., 2012). Full details regarding above mechanisms escape the scope of this article and readers are referred to a review by Martins et al. (2012). With regard to disuse induced muscle atrophy, following (7 days) bed-rest healthy volunteers showed reduced glucose infusion rate and leg glucose extraction (after bed rest) along with reduced muscle GLUT4, hexokinase II, protein kinase B/Akt1, and Akt2 protein levels, and a tendency for reduced 3-hydroxyacyl-CoA dehydrogenase activity (Biensø et al., 2012). Further in the same study, the ability of insulin to phosphorylate Akt and activate glycogen synthase was reduced post bed-rest (Biensø et al., 2012); but whether this observation is causative or a consequence of immobilization is not clear. However, a substantial decline in glucose uptake within 24 h of immobilization would argue against a causative effect (Atherton et al., 2016). Recently, Vigelso et al. showed an inverse association between the increase in muscle pyruvate dehydrogenase complex (PDC) activation and leg lactate release during contraction after 2 weeks unilateral lower limb immobilization, suggesting PDC as a potential key regulator of immobilization induced muscle IR (Vigelsø et al., 2016). Overall the above data suggests that muscle disuse results in development of whole body and muscle specific IR, fuelling the argument that lack of muscle contraction *per se* may be the main physiological driver for this dysregulation, however a mechanistic explanation for this still remains unclear (Atherton et al., 2016).

## Conclusions

Disuse muscle atrophy causes many undesirable complications. There seems to be complex interplay of numerous mechanisms contributing to the aetiology of disuse muscle atrophy. During muscle disuse, both post-absorptive and post-prandial MPS is suppressed, with little evidence to support there being an increase in “bulk” protein MPB. Moreover, animal models show increased (2.5 times) rate of muscle protein turnover and are also very sensitive to disuse, while exhibiting marked fiber-type-dependant differences in rates of muscle protein turnover (type I fibers being twice as great as type II fibers) when compared to humans. Due to these inherent species-specific differences, pre-clinical findings cannot easily be reconciled with nor extrapolated to humans. So, further research quantifying MPS and MPB and their temporal relationship during disuse in humans is warranted. There is strong evidence that type 2 diabetes accelerates muscle loss, possibly due to mechanisms innate to diabetes. Crucially, muscle IR secondary to disuse appears to drive the procession of disuse muscle atrophy independent of other mechanisms known to cause muscle IR. Nonetheless, the mechanistic role of muscle IR driving this atrophic response is poorly defined. Because, common proteolytic mechanisms may exist across “simple muscle atrophy” and other catabolic conditions (e.g., type 2 diabetes, inflammation, cachexia etc.), these two process can rarely be seen as being mutually exclusive (Atherton et al., 2016). Further, many questions remain unanswered especially the molecular regulation of MPS and MPB and muscle insulin resistance. This whole area of research has potential implications for the wider clinical community as similar metabolic processes occur during cancer cachexia, metabolic syndromes including type 2 diabetes, aging (i.e., sarcopenia), sepsis and many neurodegenerative disorders. Henceforth, further translational research is necessary before this knowledge can be effectively applied in developing targeted strategies to prevent this in the setting of disuse muscle atrophy.

## Author contributions

All authors listed, have made substantial, direct and intellectual contribution to the work, and approved it for publication.

### Conflict of interest statement

The authors declare that the research was conducted in the absence of any commercial or financial relationships that could be construed as a potential conflict of interest.
